# Supplementary material for: OsNF-YB7 inactivates OsGLK1 to inhibit chlorophyll biosynthesis in rice embryo
Source: eLife. 2024 Sep 17;13:RP96553. doi: 10.7554/eLife.96553 (PMC11407766; doi:10.7554/eLife.96553)
Supplement: Figure 2—source data 1. [file elife-96553-fig2-data1.zip › Figure 2-Source data 1/Figure 2-Source data 2 Uncropped and labeled gels for Figure 2.pdf]

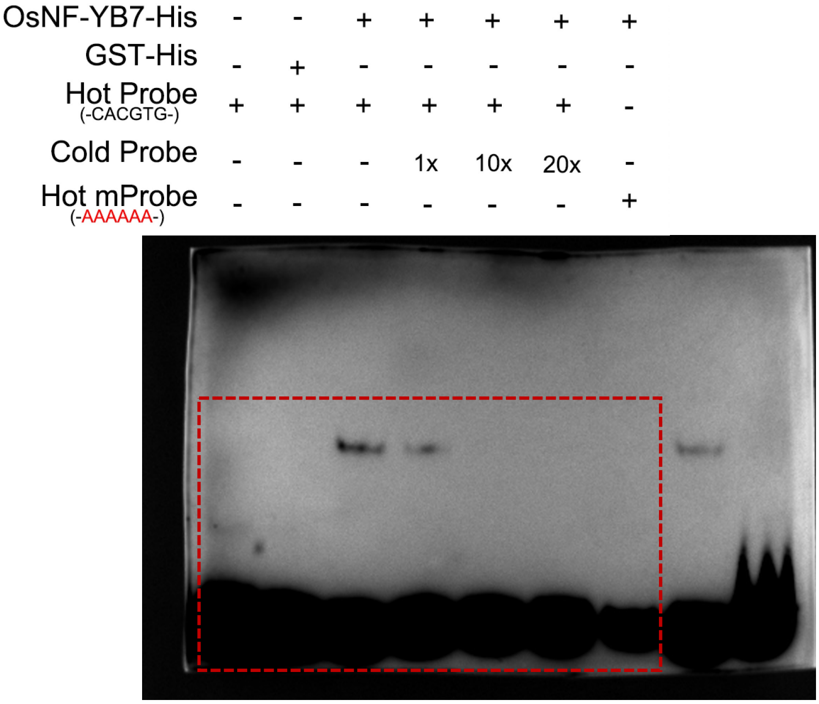

OsNF-YB7-His - - + + + + +
GST-His - + - - - - -
Hot Probe (-CACGTG-) + + + + + + -
Cold Probe - - - 1x 10x 20x -
Hot mProbe (-AAAAAA-) - - - - - - +
