# Supplementary figures and images for: OsNF-YB7 inactivates OsGLK1 to inhibit chlorophyll biosynthesis in rice embryo
Source: eLife. 2024 Sep 17;13:RP96553. doi: 10.7554/eLife.96553 (PMC11407766; doi:10.7554/eLife.96553)

|                          |   |   |    |    |    |   |   |
|--------------------------|---|---|----|----|----|---|---|
| OsNF-YB7-His             | - | + | +  | +  | +  | + | - |
| GST-His                  | - | - | -  | -  | -  | - | + |
| Hot Probe<br>(-CACGTG-)  | + | + | +  | +  | +  | - | + |
| Cold Probe               | - | - | 1x | 2x | 5x | - | - |
| Hot mProbe<br>(-AAAAAA-) | - | - | -  | -  | -  | + | - |

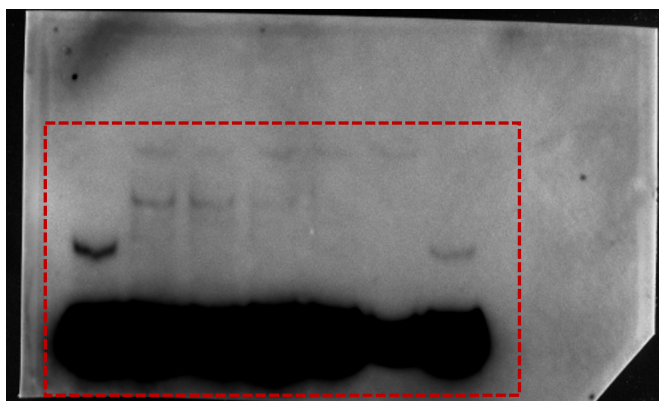

Supplement: Figure 2—source data 1. [file elife-96553-fig2-data1.zip › Figure 2-Source data 1/Figure 2-Source data 1 Uncropped and labeled gels for Figure 2.pdf]

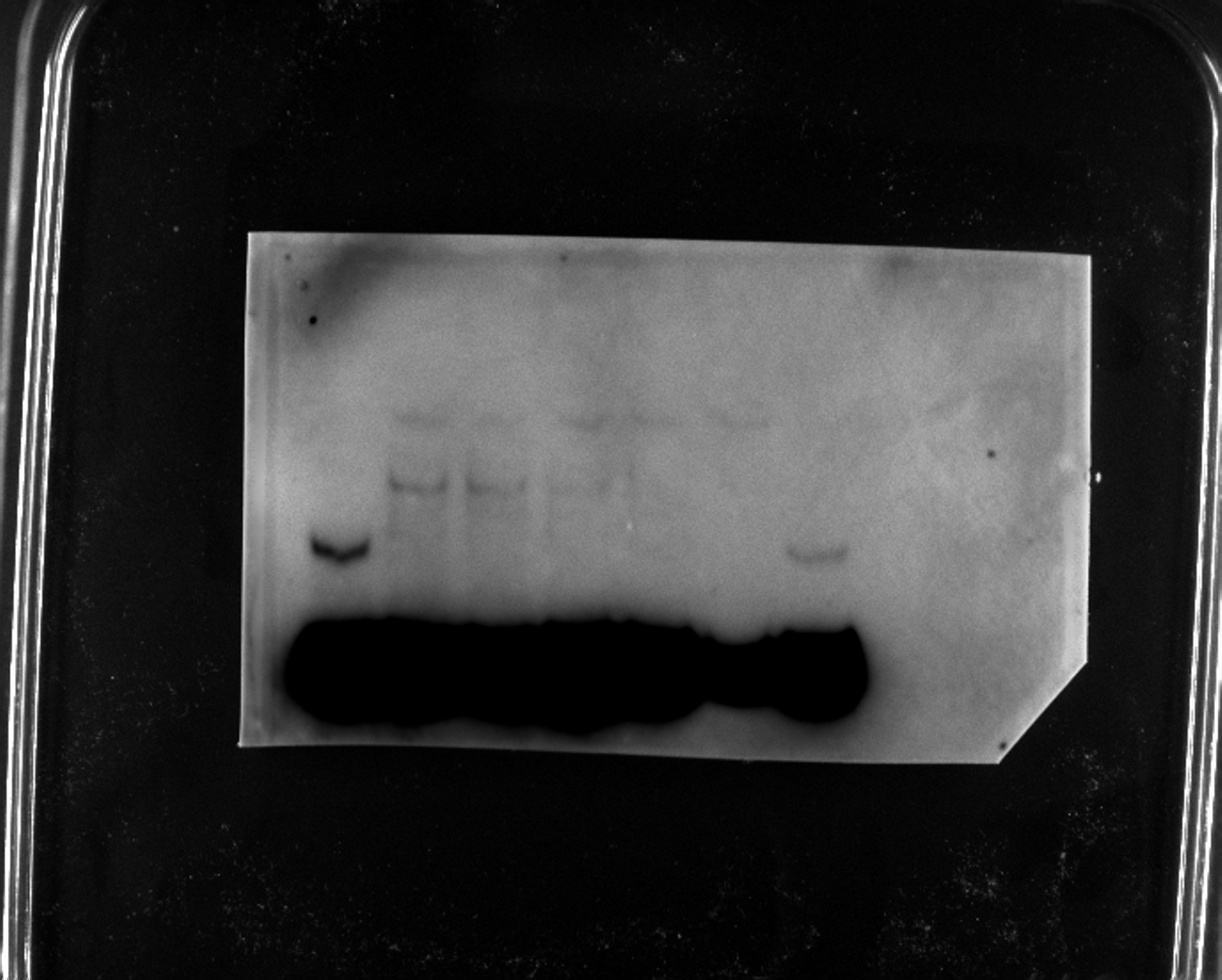

Supplement: Figure 2—source data 2. [file elife-96553-fig2-data2.zip › Figure 2-Source data 2/Figure 2-source data 1 Raw unedited gels for Figure 2.jpg]

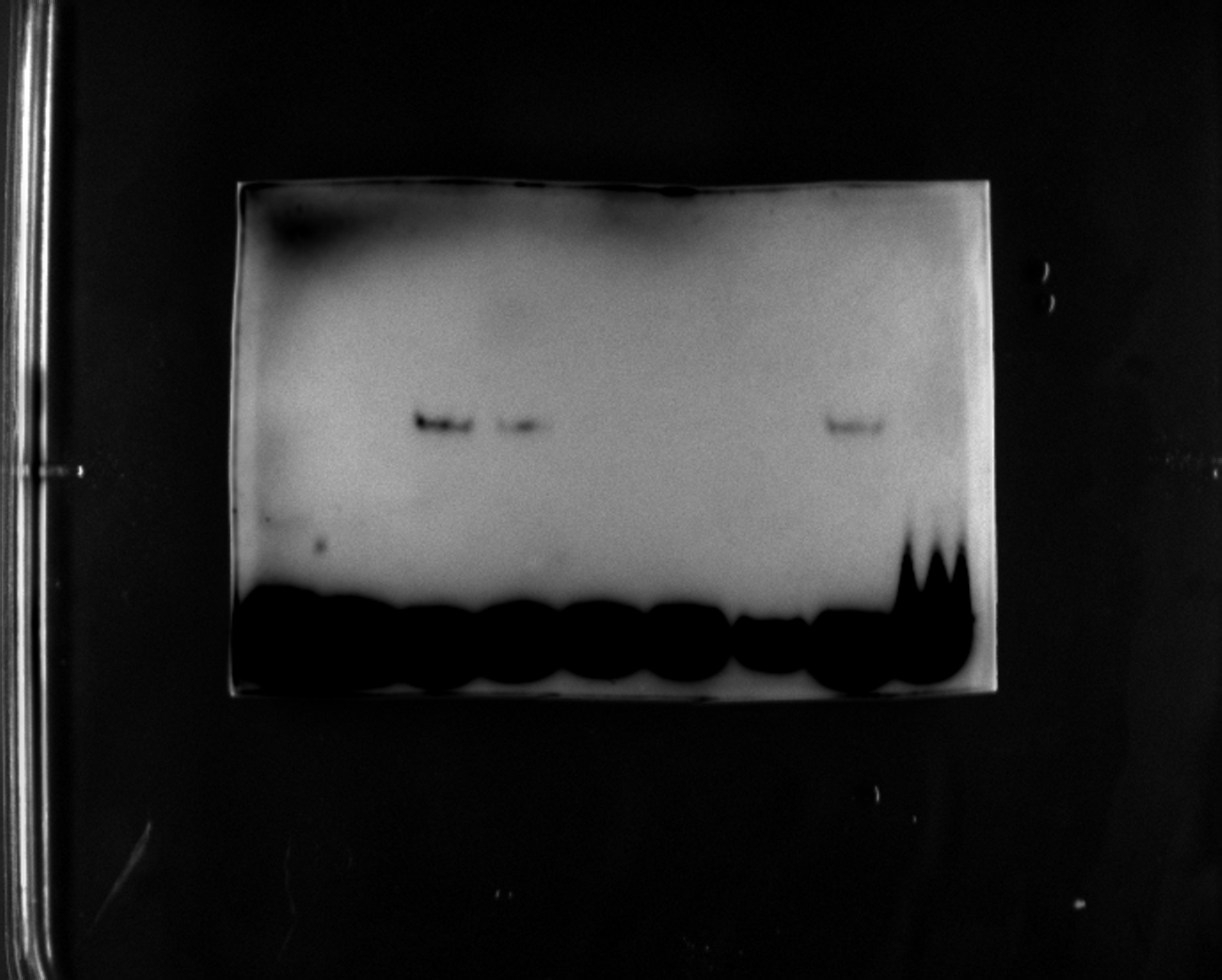

Supplement: Figure 2—source data 2. [file elife-96553-fig2-data2.zip › Figure 2-Source data 2/Figure 2-source data 2 Raw unedited gels for Figure 2.jpg]

|                     |   |   |     |   |
|---------------------|---|---|-----|---|
| OsNF-YB7-His        | - | + | +   | + |
| Hot Probe (OsGLK1)  | + | + | +   | - |
| Cold Probe (OsGLK1) | - | - | 50x | - |
| Hot Probe (OsLHCB4) | - | - | -   | + |

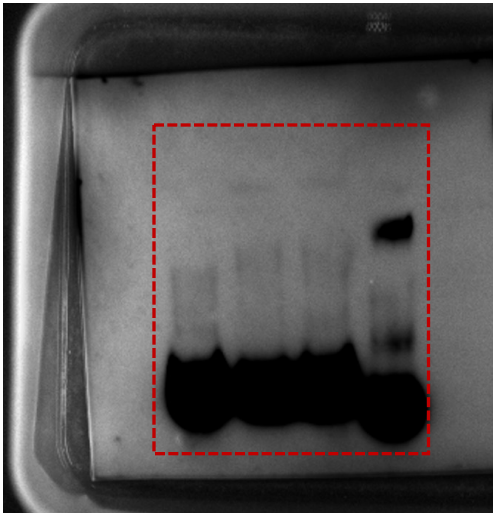

Supplement: Figure 3—figure supplement 1—source data 1. [file elife-96553-fig3-figsupp1-data1.zip › Figure 3ΓÇôfigure supplement 1-source data 1/Figure 3ΓÇôfigure supplement 1-source data 1 Uncropped and labeled gels for Figure 3ΓÇôfigure supplement 1.pdf]

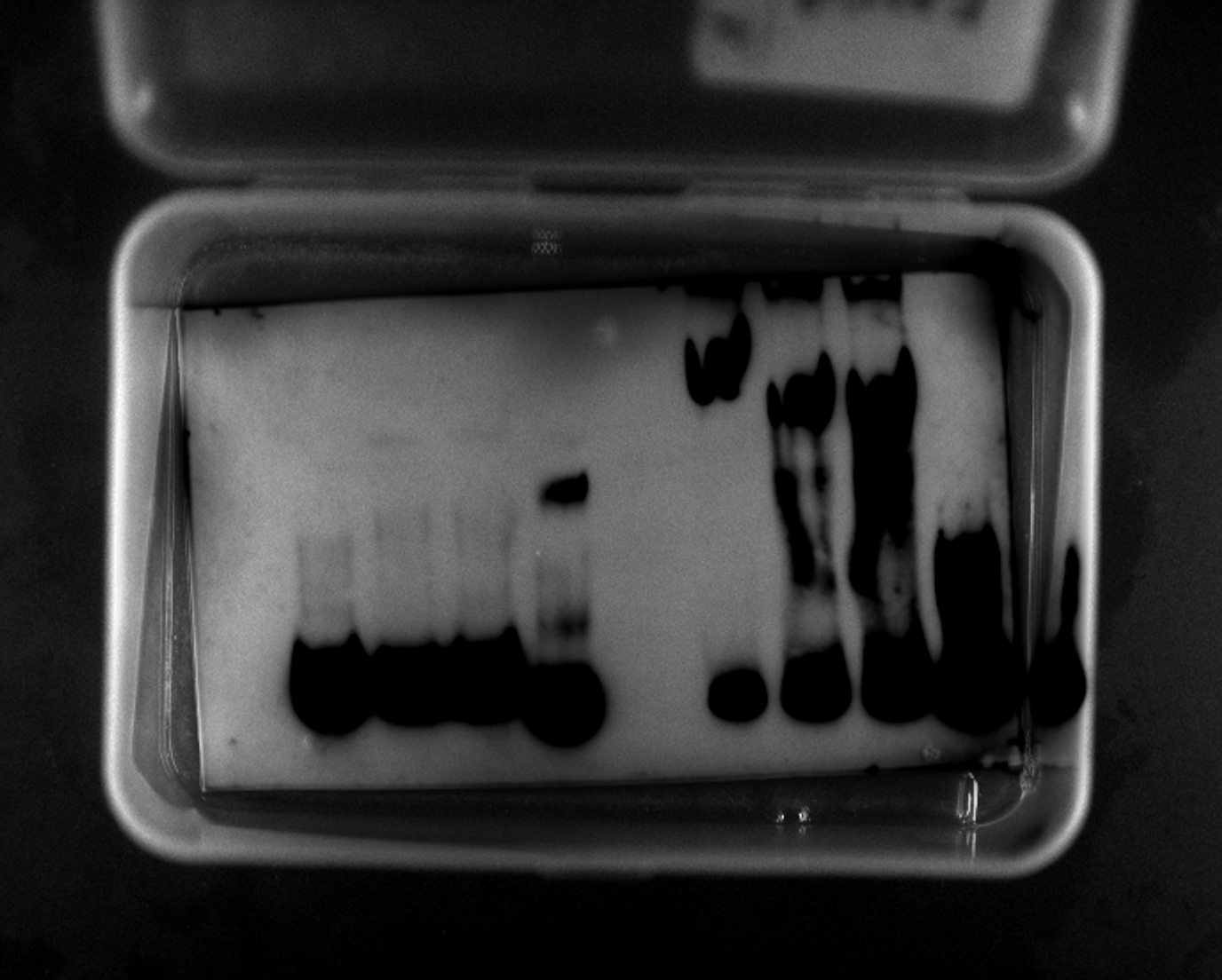

Supplement: Figure 3—figure supplement 1—source data 2. [file elife-96553-fig3-figsupp1-data2.zip › Figure 3ΓÇôfigure supplement 1-source data 2/Figure 3ΓÇôfigure supplement 1-source data 1 Raw unedited gels for Figure 3ΓÇôfigure supplement 1.jpg]

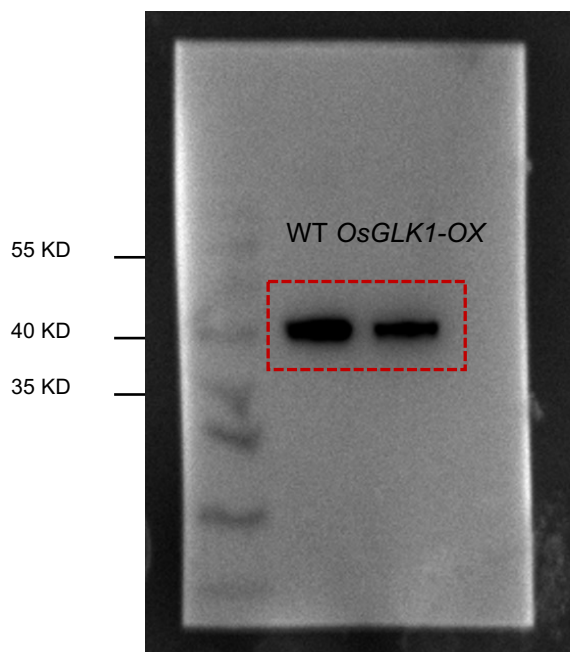

$\alpha$ -GAPDH

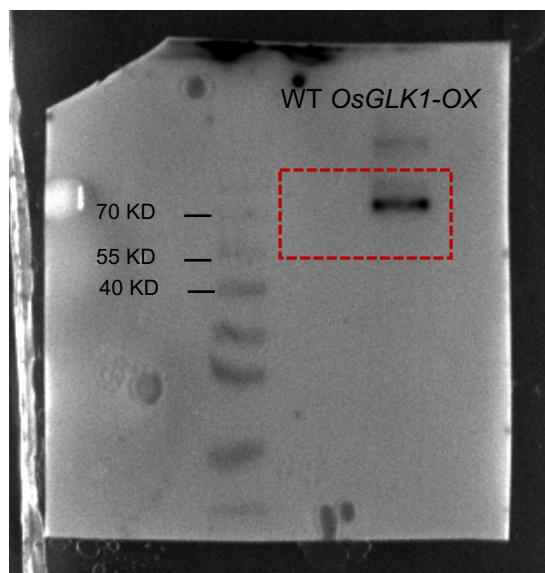

$\alpha$ -Flag

Supplement: Figure 4—figure supplement 1—source data 1. [file elife-96553-fig4-figsupp1-data1.zip › Figure 4ΓÇôfigure supplement 1-source data 1/Figure 4ΓÇôfigure supplement 1-source data 1 Uncropped and labeled gels for Figure 4ΓÇôfigure supplement 1.pdf]

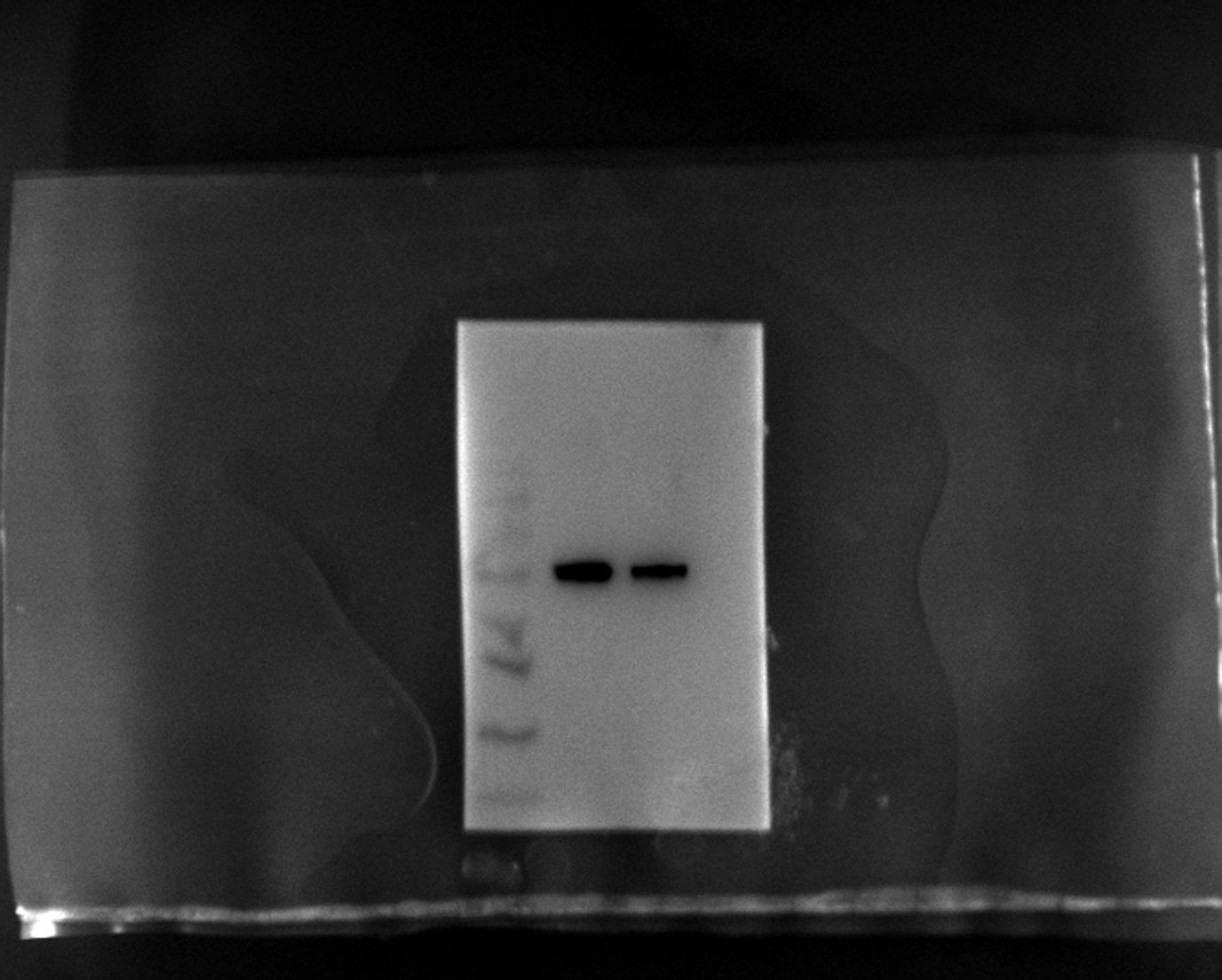

Supplement: Figure 4—figure supplement 1—source data 2. [file elife-96553-fig4-figsupp1-data2.zip › Figure 4ΓÇôfigure supplement 1-source data 2/Figure 4ΓÇôfigure supplement 1-source data 1 Raw unedited gels for Figure 4ΓÇôfigure supplement 1.jpg]

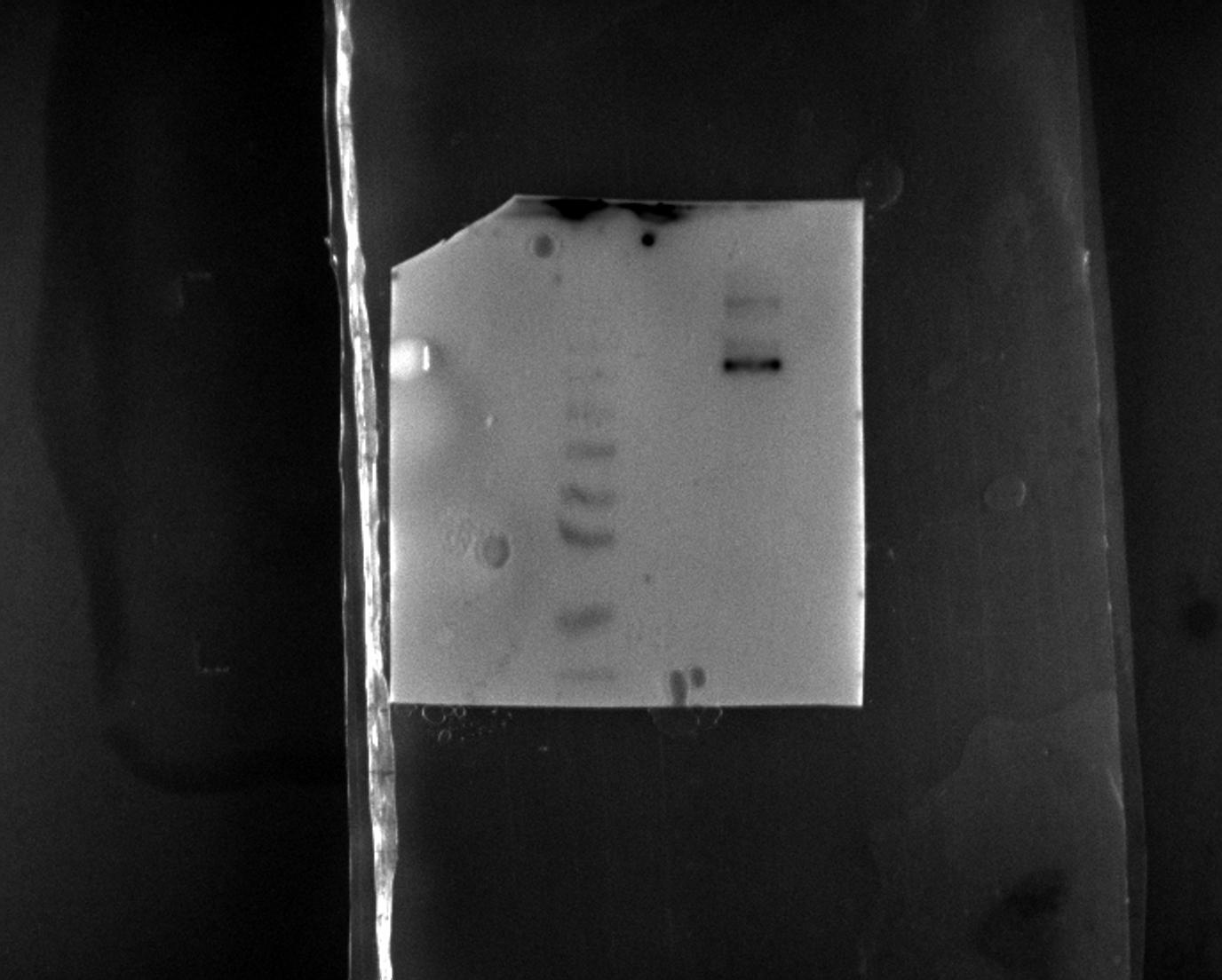

Supplement: Figure 4—figure supplement 1—source data 2. [file elife-96553-fig4-figsupp1-data2.zip › Figure 4ΓÇôfigure supplement 1-source data 2/Figure 4ΓÇôfigure supplement 1-source data 2 Raw unedited gels for Figure 4ΓÇôfigure supplement 1.jpg]

|            | P2 |   |     |      |      |   |
|------------|----|---|-----|------|------|---|
| OsGLK1-MBP | -  | + | +   | +    | +    | - |
| MBP        | -  | - | -   | -    | -    | + |
| Hot Probe  | +  | + | +   | +    | +    | + |
| Cold Probe | -  | - | 50x | 100x | 200x | - |

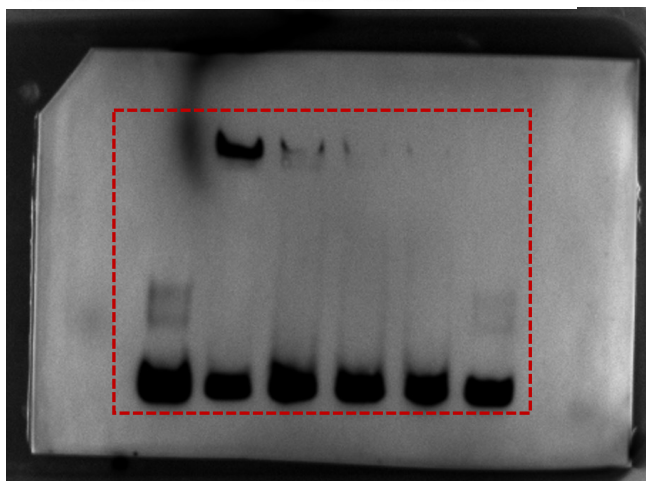

Supplement: Figure 5—source data 2. [file elife-96553-fig5-data2.zip › Figure 5-Source data 2/Figure 5-Source data 1 Uncropped and labeled gels for Figure 1.pdf]

|            | P3 |   |     |     |     |   |
|------------|----|---|-----|-----|-----|---|
| OsGLK1-MBP | -  | + | +   | +   | +   | - |
| MBP        | -  | - | -   | -   | -   | + |
| Hot Probe  | +  | + | +   | +   | +   | + |
| Cold Probe | -  | - | 10x | 20x | 50x | - |

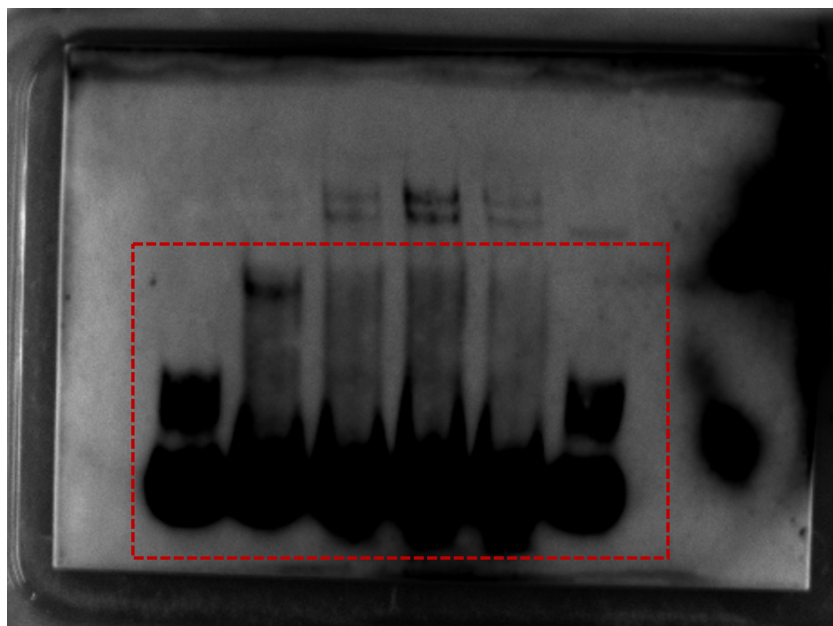

Supplement: Figure 5—source data 2. [file elife-96553-fig5-data2.zip › Figure 5-Source data 2/Figure 5-Source data 1 Uncropped and labeled gels for Figure 2.pdf]

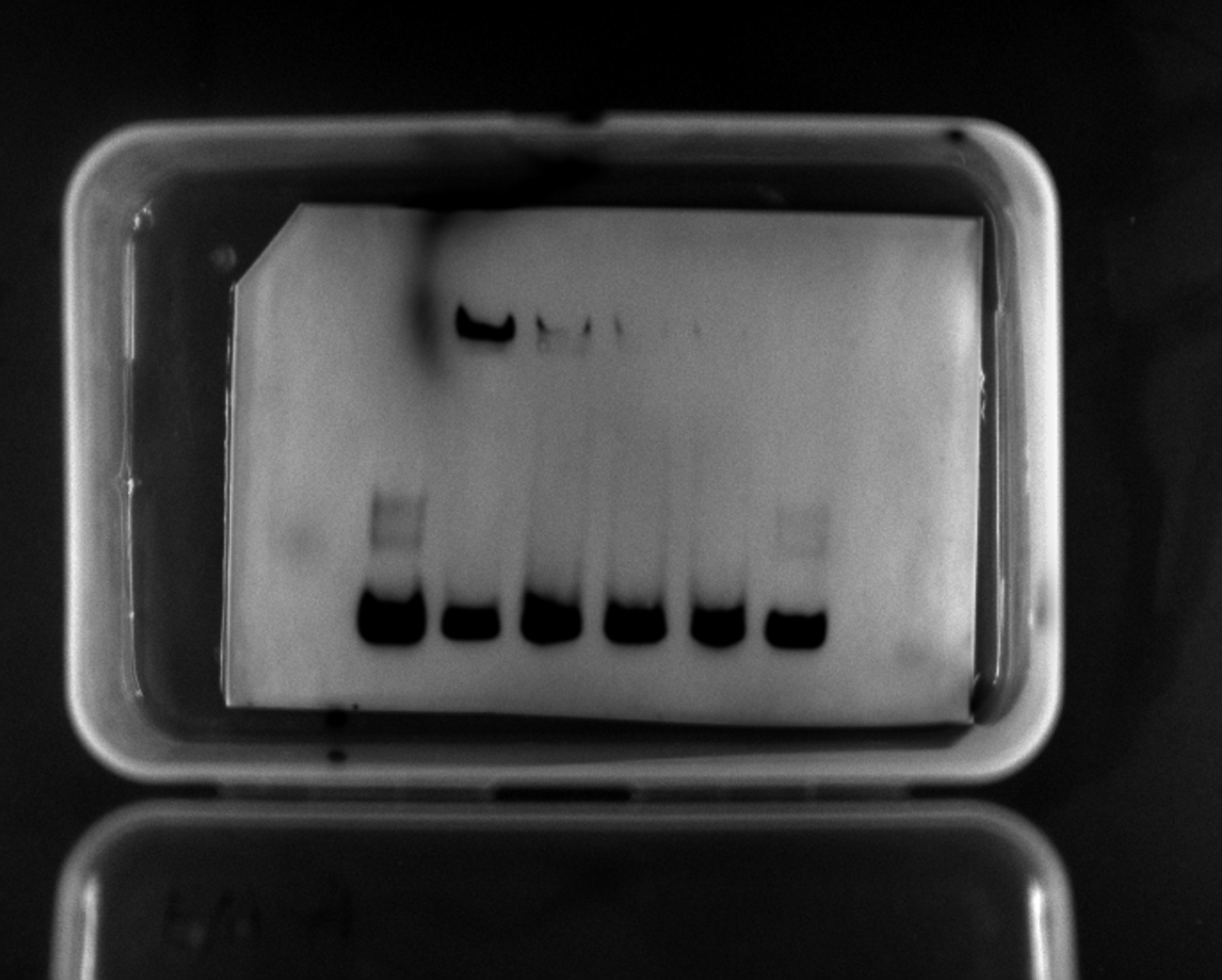

Supplement: Figure 5—source data 3. [file elife-96553-fig5-data3.zip › Figure 5-Source data 3/Figure 5-source data 1 Raw unedited gels for Figure 5.jpg]

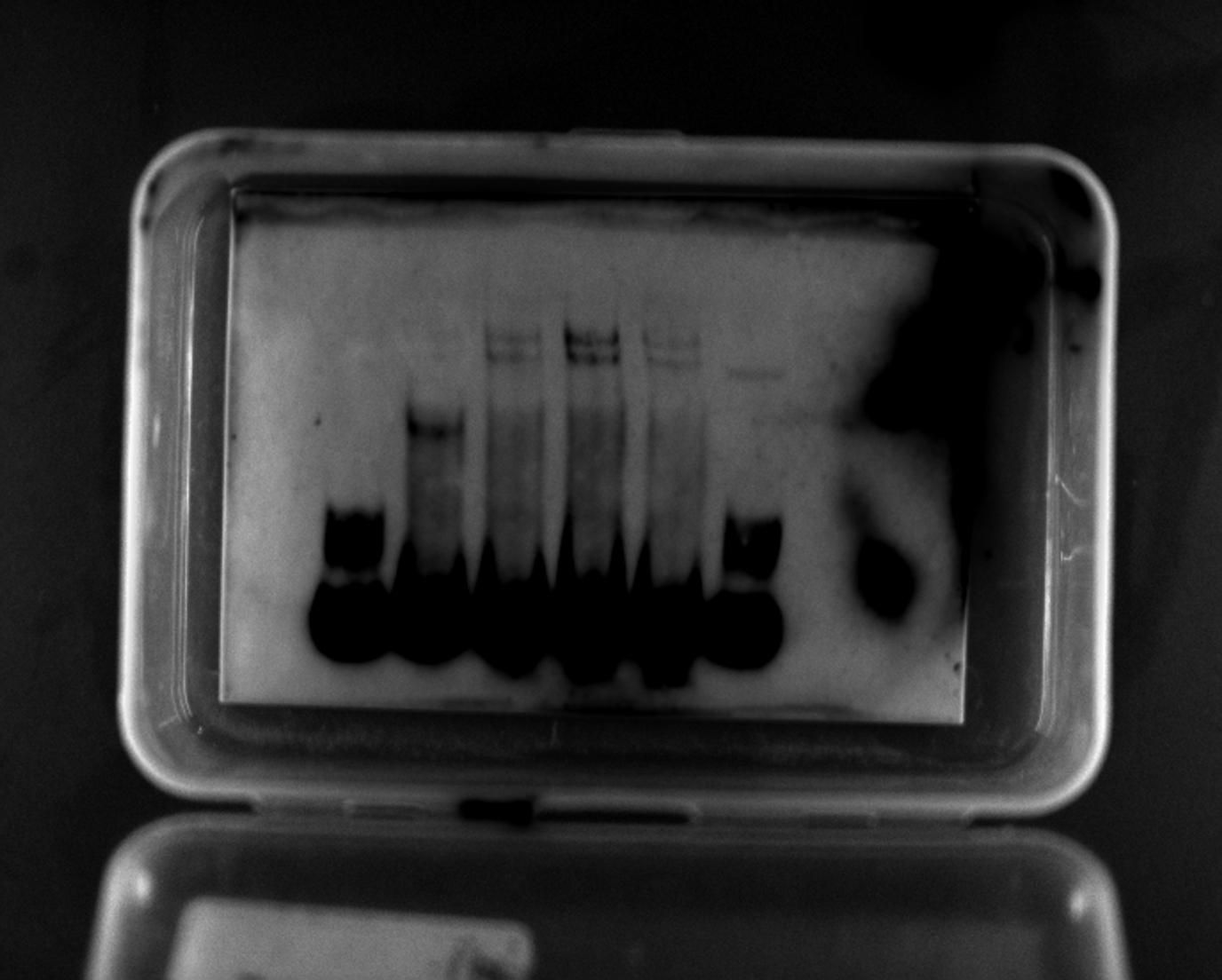

Supplement: Figure 5—source data 3. [file elife-96553-fig5-data3.zip › Figure 5-Source data 3/Figure 5-source data 2 Raw unedited gels for Figure 5.jpg]

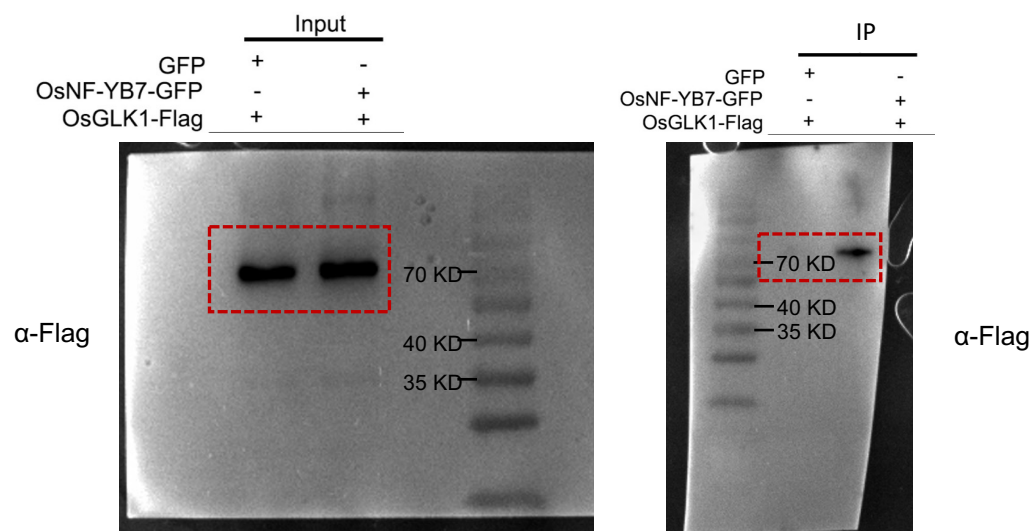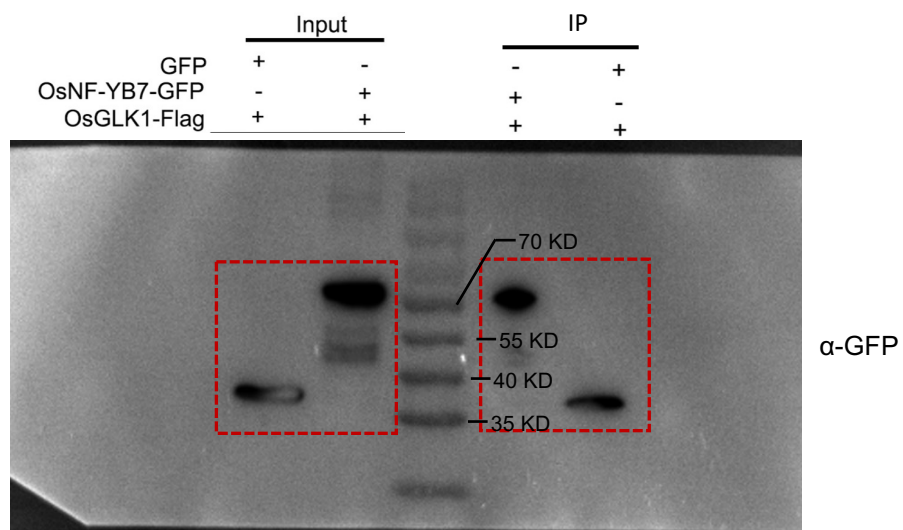

Supplement: Figure 6—source data 1. [file elife-96553-fig6-data1.zip › Figure 6-Source data 1/Figure 6-Source data 1 Uncropped and labeled gels for Figure 6.pdf]

|              |   |   |   |   |   |   |    |    |
|--------------|---|---|---|---|---|---|----|----|
| Probe        | + | + | + | + | + | + | +  | +  |
| OsNF-YB7-His | - | - | - | + | - | + | ++ | -  |
| OsGLK1-MBP   | - | - | - | - | + | + | +  | +  |
| MBP          | - | - | + | - | - | - | -  | -  |
| GST-His      | - | + | - | - | - | - | -  | ++ |

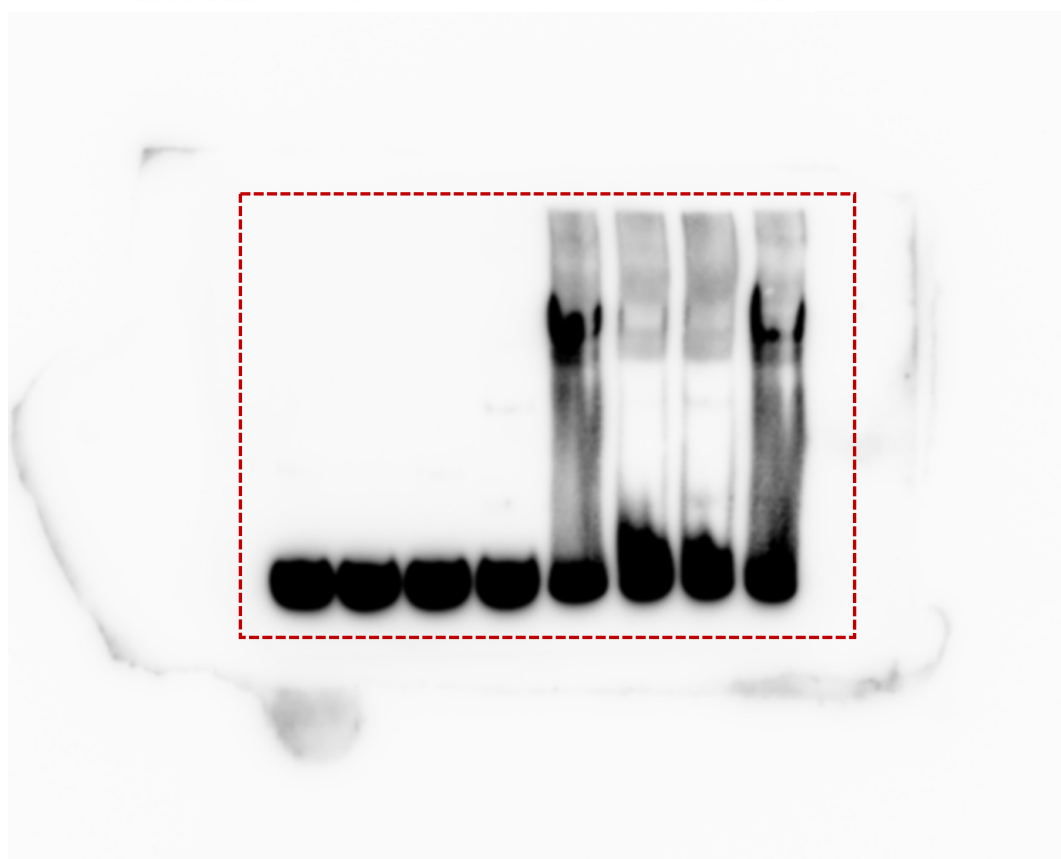

Supplement: Figure 6—source data 1. [file elife-96553-fig6-data1.zip › Figure 6-Source data 1/Figure 6-Source data 2 Uncropped and labeled gels for Figure 6.pdf]

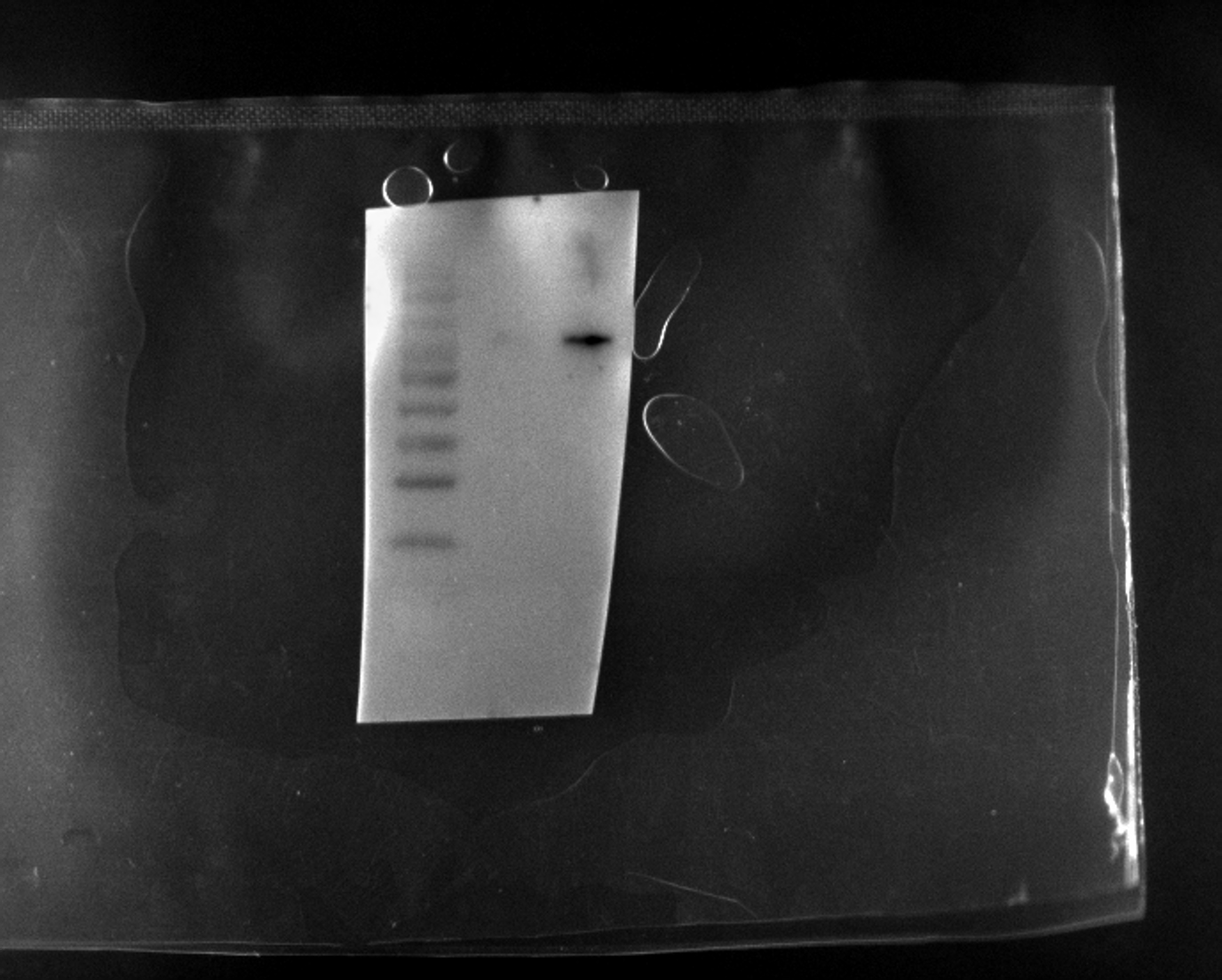

Supplement: Figure 6—source data 2. [file elife-96553-fig6-data2.zip › Figure 6-Source data 2/Figure 6-source data 3 Raw unedited gels for Figure 6.jpg]

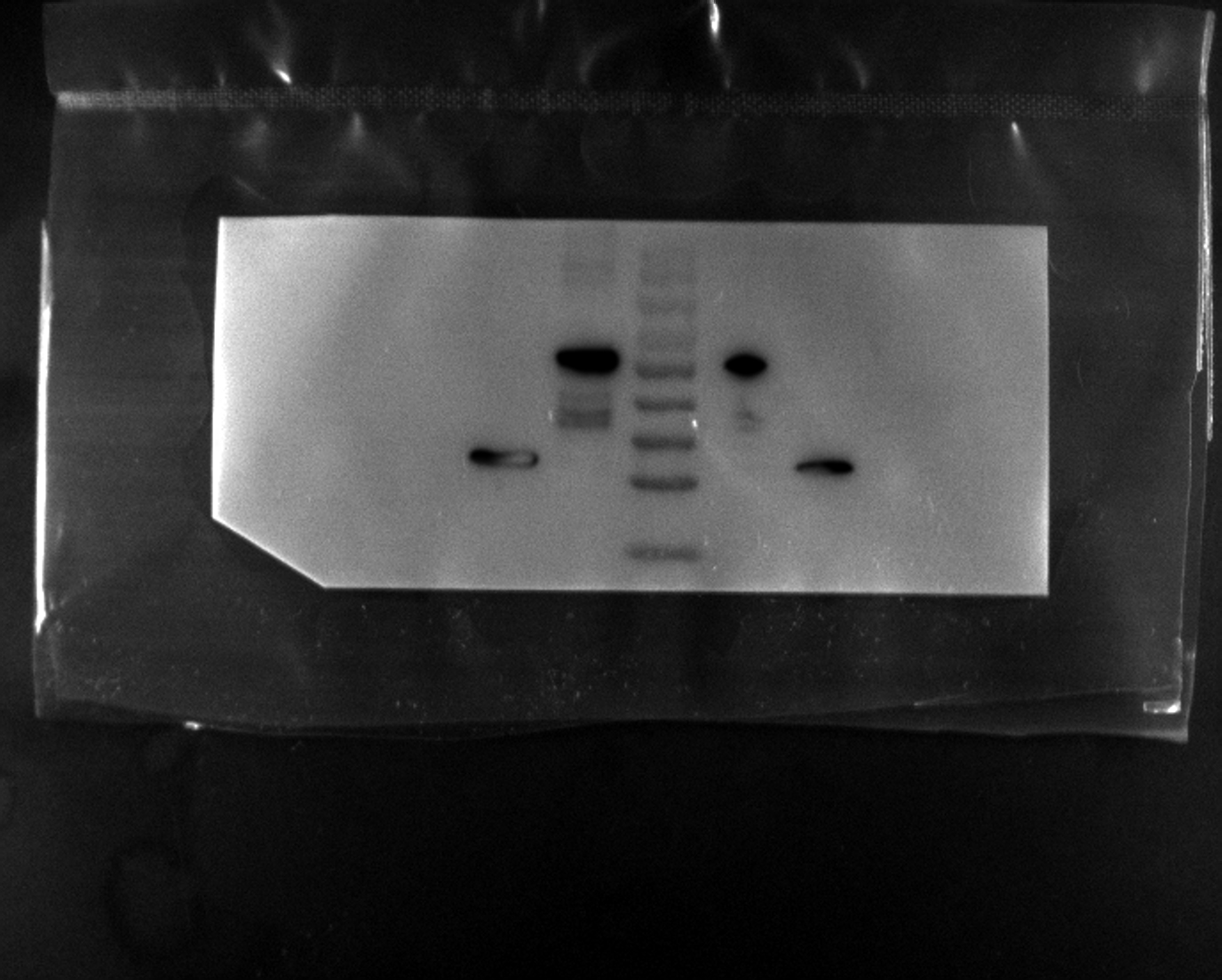

Supplement: Figure 6—source data 2. [file elife-96553-fig6-data2.zip › Figure 6-Source data 2/Figure 6-source data 2 Raw unedited gels for Figure 6.jpg]

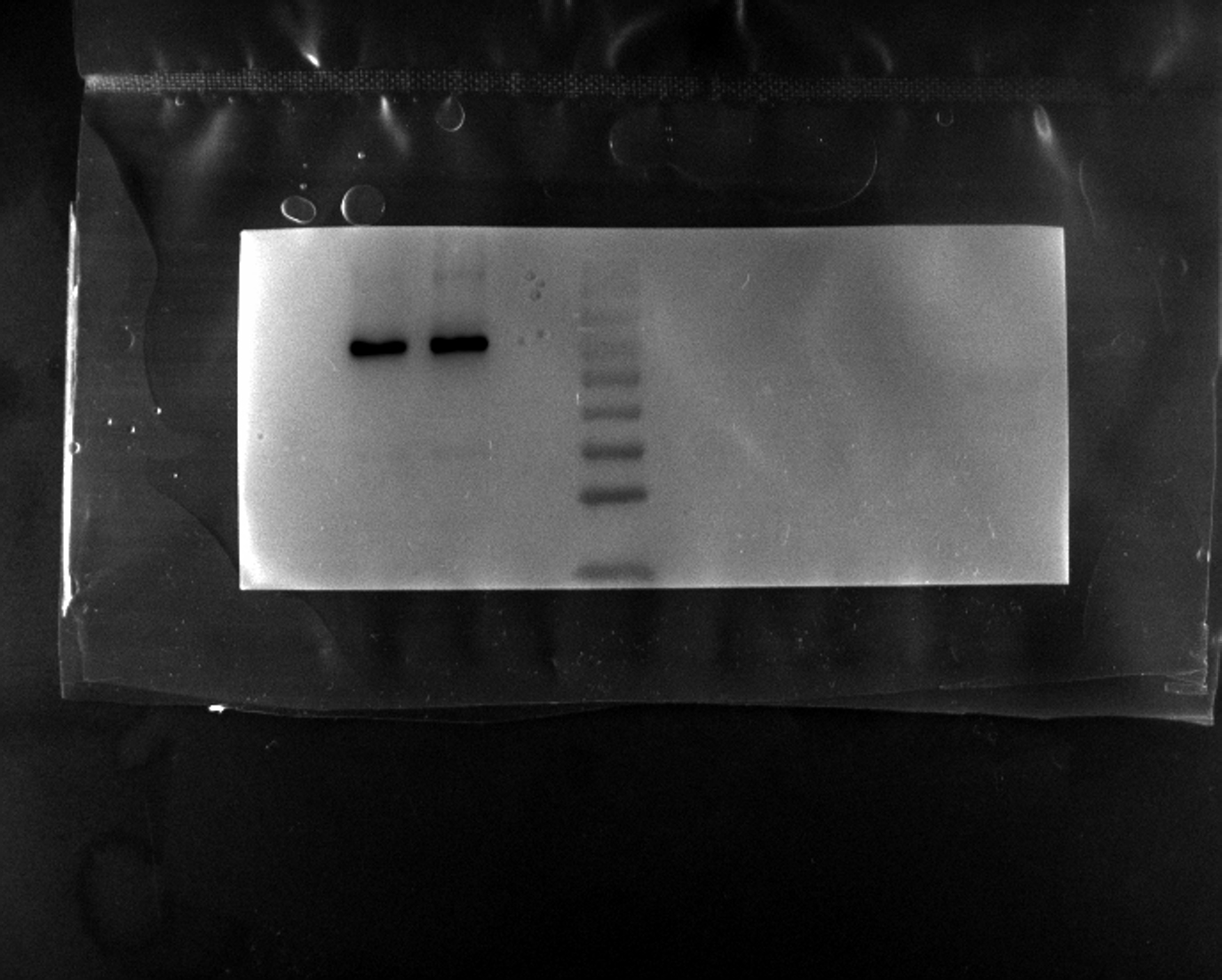

Supplement: Figure 6—source data 2. [file elife-96553-fig6-data2.zip › Figure 6-Source data 2/Figure 6-source data 1 Raw unedited gels for Figure 6.jpg]

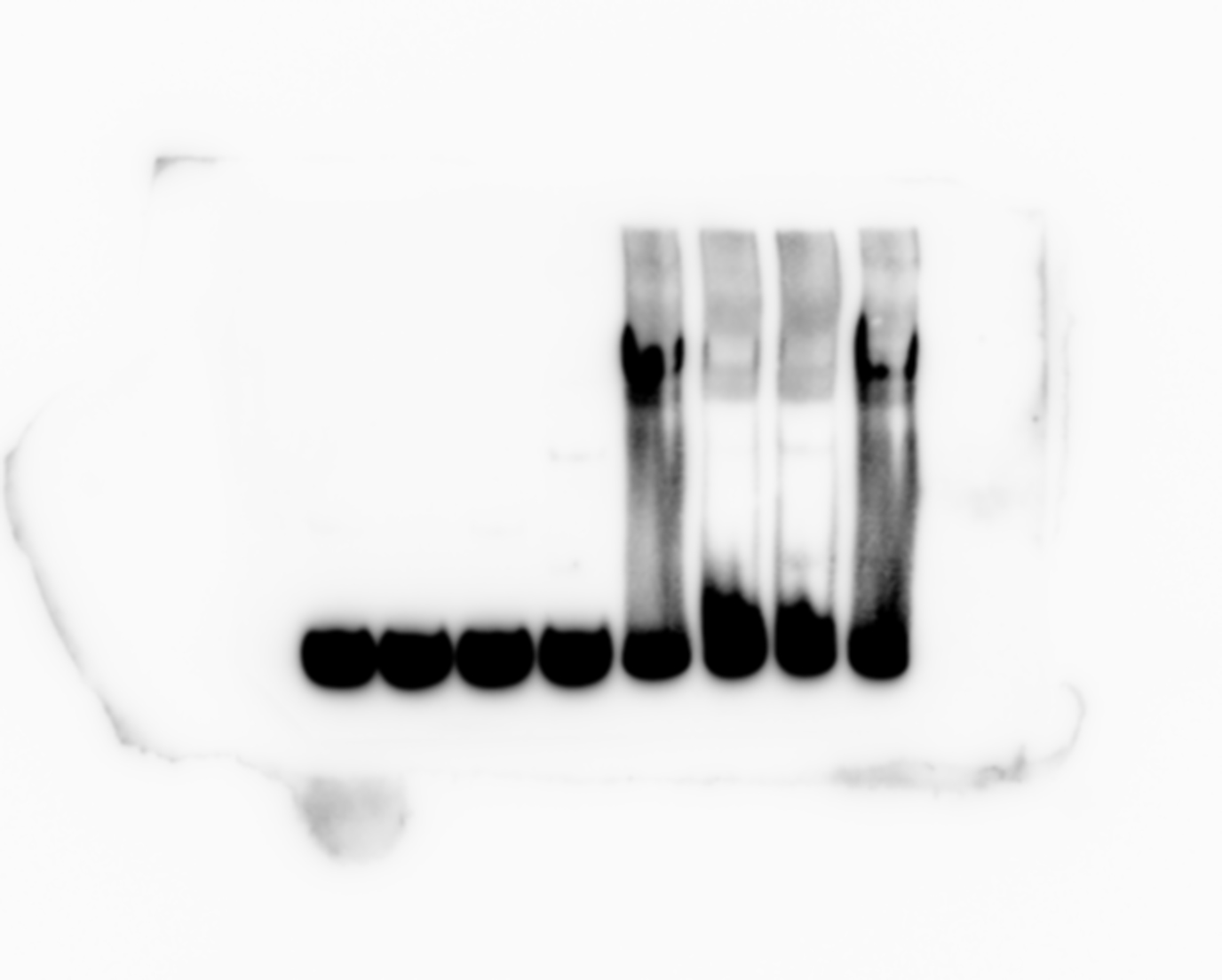

Supplement: Figure 6—source data 2. [file elife-96553-fig6-data2.zip › Figure 6-Source data 2/Figure 6-source data 4 Raw unedited gels for Figure 6.jpg]
